# Supplementary material for: Chronic Electrical Stimulation of the Superior Laryngeal Nerve in the Rat: A Potential Therapeutic Approach for Postmenopausal Osteoporosis
Source: Biomedicines. 2020 Sep 22;8(9):369. doi: 10.3390/biomedicines8090369 (PMC7555126; doi:10.3390/biomedicines8090369)
Supplement: Supplementary file 1 [file biomedicines-08-00369-s001.pdf]

Supplementary materials

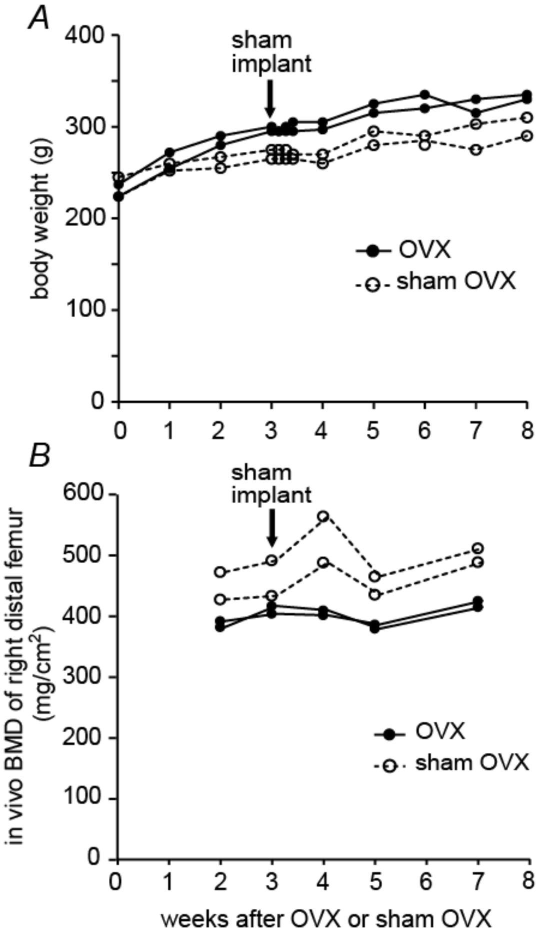

Figure S1. Changes in body weight and bone mineral density (BMD) following ovariectomy (OVX). Closed circles indicate data of OVX rats (n = 2) and open circles indicate data of sham OVX rats (n = 2). At 3 weeks after OVX, as the sham implant, only head socket was fixed on the skull without cuff electrode implantation. Body weight at 0 week was obtained on the day of OVX (prior to surgery) and measured weekly until 8 weeks later (**A**). Also body weight was monitored for consecutive 4 days (on the day and following sham implant surgery). BMD of right distal femur was obtained by radioabsometry using in vivo imaging system (in-vivo Xtreme. Bruker BioSpin, Billerica, ME) starting at 2 weeks after OVX and onwards (**B**). On the day of imaging, rats were anesthetized with isoflurane (1-4%) and the tether removed from the head socket. Then, rats were placed into the imaging device in a prone position, with a face mask to maintain the anesthesia (1-2%). The X-ray image of the right thigh was acquired taking for about 1 min. For BMD quantification, the same location of femur was chosen for analysis as dual energy X-ray absorptiometry (DXA) (Figure 4).

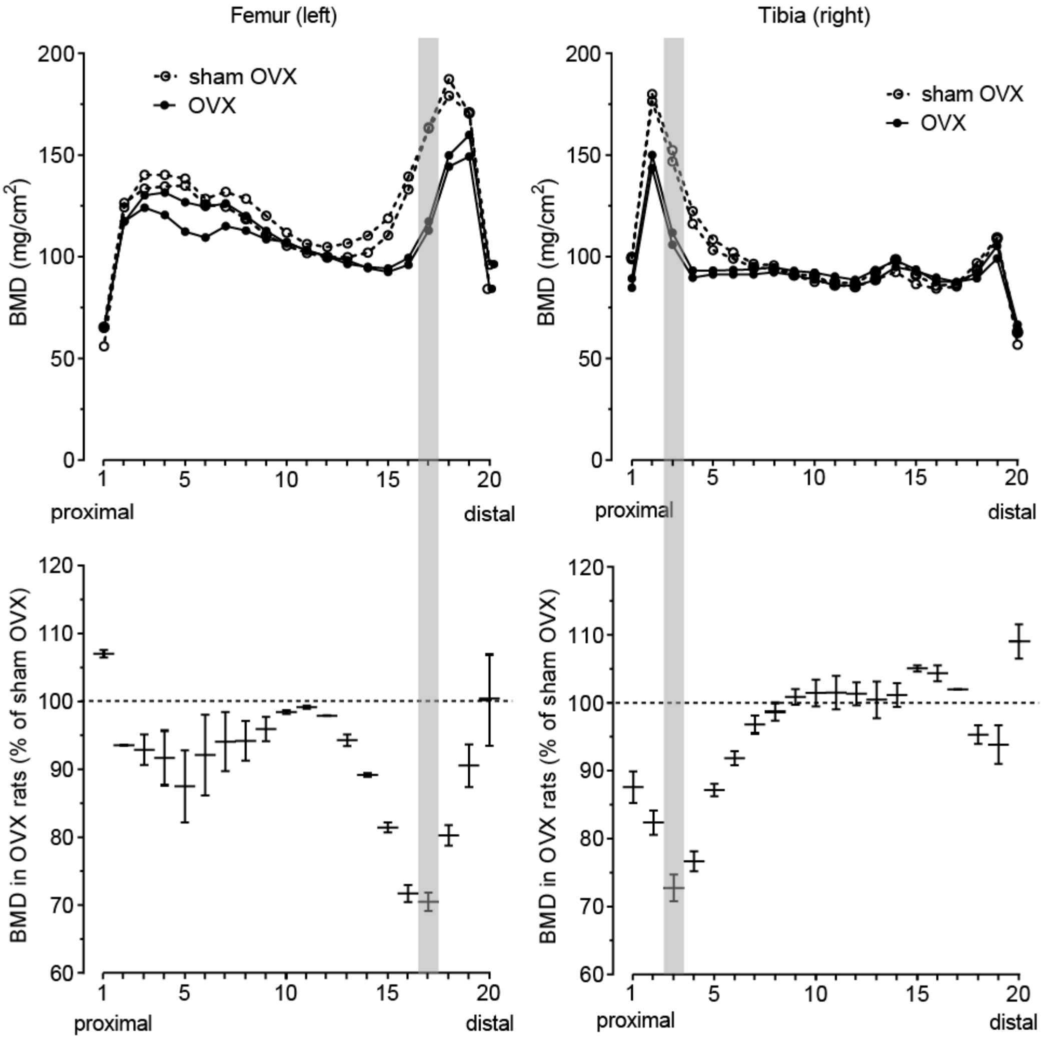

Figure S2. The influence of ovariectomy (OVX) on bone mineral density (BMD) in femur and tibia. BMD was obtained using the dual energy X-ray absorptiometry (DXA) method. Upper graphs show BMD in OVX and sham OVX rats. The whole bone was equally divided into 20 parts along the long axis. Circles represent BMD obtained at each point. Closed circles indicate data of OVX rats and open circles indicate data of sham OVX rats. Lower graphs show changes in BMD in OVX rats expressed as % of average value of sham OVX rats. The severest reduction was noted at metaphysis of distal femur (4th section from distal end) and that of proximal tibia (3rd section from proximal end), highlighted by vertical gray bars.

Table S1. Influence of ovariectomy (OVX) on bone mineral density (BMD), bone strength and bone histomorphometry

	Unit	Sham OVX (n = 2)		OVX (n = 2)	
		Rat 1	Rat 2	Rat 3	Rat 4
**DXA-BMD**					
proximal tibia	mg/cm^2	152.4	146.9	105.9	111.8
distal femur	mg/cm^2	163.0	163.6	112.9	117.3
**Bone strength*** [breaking force]	N	119.2	129.3	122.3	117.3
**Bone histomorphometry**					
**Bone structure**					
Cancellous bone volume	%	35.5	27.9	6.6	8.8
Trabecular thickness	μm	63.7	65.5	59.1	61.2
Trabecular number	per mm	5.6	4.3	1.1	1.4
**Osteogenesis**					
Osteoid surface	%	0.5	0.8	12.6	8.1
Osteoblast surface	%	0.9	0.4	8.8	3.3
Bone calcification surface	%	3.1	5.8	22.7	19.4
Calcification rate	μm/day	1.2	1.1	1.1	1.4
**Bone resorption**					
Erosional surface	%	2.5	0.7	8.8	8.0
Osteoclast surface	%	1.3	0.4	3.7	3.5
Osteoclast number	per 100mm	96.8	39.5	198.6	175.7

DXA; dual energy X-ray absorption

* Measured by the three-point bending test (the femur were fixed and vertical force was applied to the center of the femur diaphysis until the bone breaks).
